# Advances and Challenges in Bioprocess Optimization for the Synthesis of Sugar Nucleotides

**DOI:** 10.1002/cbic.202500434

**Published:** 2025-10-03

**Authors:** Tom L. Roberts, Sebastian C. Cosgrove, Gavin J. Miller

**Affiliations:** ^1^ Centre for Glycoscience and School of Chemical and Physical Sciences Keele University Keele Staffordshire ST5 5BG UK

**Keywords:** biocatalysis, bioprocess, glycan, immobilisation, sugar nucleotide

## Abstract

Sugar nucleotides represent the cornerstone building blocks for glycan biosynthesis. While methods to access these crucial biomolecules using traditional batch synthetic chemistry and enzymatic approaches have blossomed, uptake using flow‐based synthesis is burgeoning. This perspective analyzes recent advances concerning enzyme immobilization and continuous flow biocatalysis for sugar nucleotide production and usage. Evaluation of related technologies is also discussed, highlighting new enzyme immobilization approaches, novel reactor design, and improved downstream processing as areas that must evolve to enable wider, scalable access to sugar nucleotides as commodity chemicals.

## Graphical abstract

The emergence of flow biocatalysis for the production of glycan building blocks, sugar nucleotides, is considered.

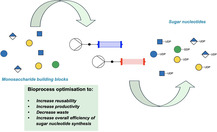

## 1 Introduction

Glycans are produced by all living organisms and are an essential group of macromolecules that coat the surface of cells and form an integral part of the intracellular matrix.^[^ ^1^ ^,^ ^2^ ^]^ Many glycans are thus positioned to modulate or mediate a plethora of biological processes, yet unlike translation and transcription, glycan biosynthesis is not templated which, in addition to the vast range of monosaccharide building blocks available, results in a far greater combinatorial glycan diversity when compared to the macromolecules derived from nucleosides or amino acids. As a result of this biological ubiquity, there is a sustained scientific interest around the synthesis and utilization of glycan structures.

Within biological glycan synthesis, sugar nucleotides (activated sugar donors) are utilized in glycosylation‐based biosynthetic pathways in conjunction with a diverse range of acceptors, including other glycans, proteins, and lipids, releasing an energetic mono‐ or dinucleotide byproduct. As such, these biomolecules have been the focus of much interest from communities interested in constructing and utilizing them, often as tools to study related biological processes or as building blocks for wider glycan assembly. Synthetic chemistry and enzymatic techniques to assemble sugar nucleotides have strong foundations, and their unification may well be transformative in addressing the overall challenge of rapidly accessing such motifs.^[^ ^3^, ^4^ ^–^ ^5^ ^]^

In this perspective, we consider the emergent area of bioprocess development for enzymatic sugar nucleotide synthesis, drawn from progress in the wider field of sustainable biocatalysis,^[^ ^6^ ^]^ and include examples surrounding immobilization of enzymes and applications in continuous flow alongside consideration of enzyme cofactors. These developments will necessarily underpin a wider (and transformative) access/uptake for these building blocks that are central to the assembly of carbohydrate‐containing biomolecules; we conclude by considering current challenges in this bioprocess development space.

## 2 Sugar Nucleotide Synthesis and Utilization in Continuous Flow Biocatalysis

### 2.1 Soluble Enzymes

**Figure 1:**
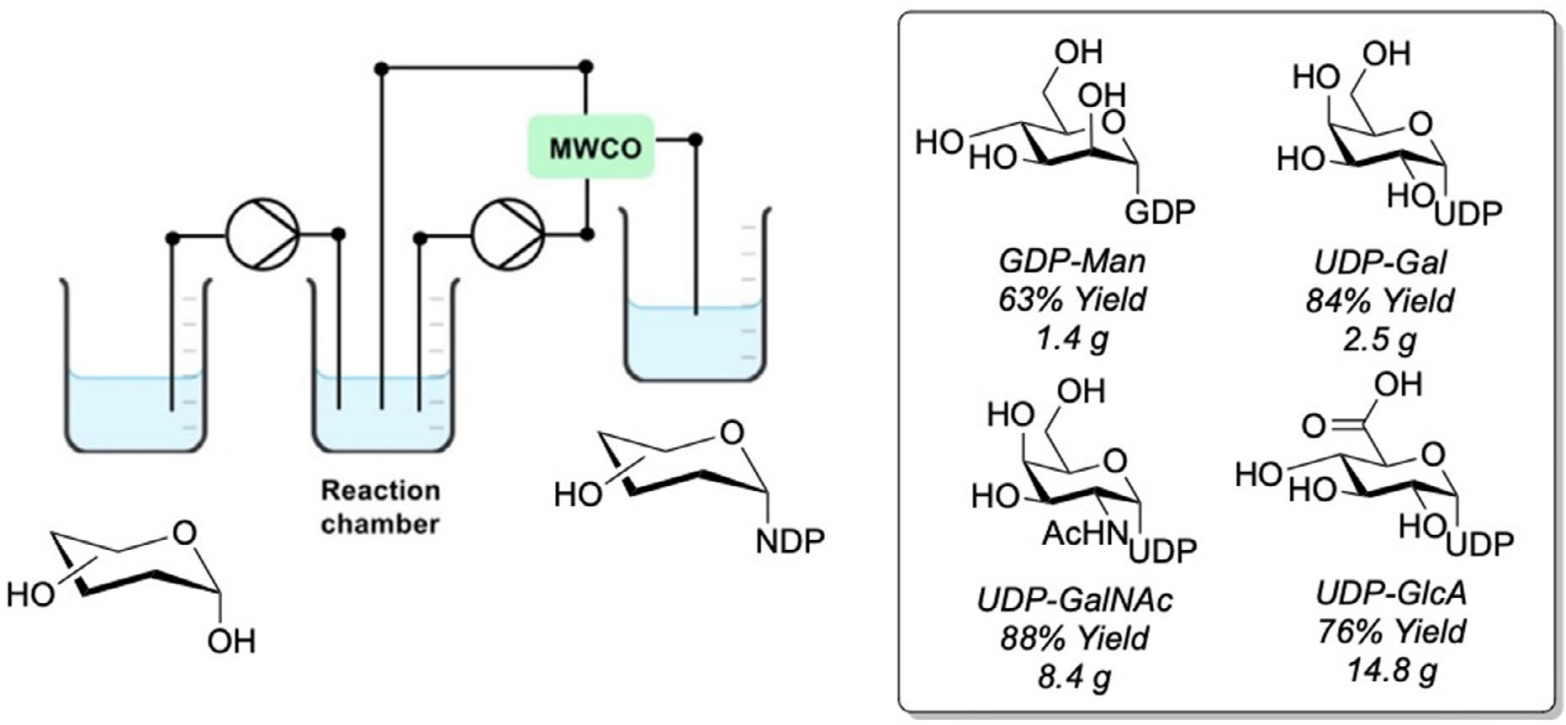
Compartmentalized reactor setup with MWCO filter to retain soluble enzymes for multigram scale synthesis of sugar nucleotides. In the case of UDP‐GlcA synthesis, AtGlcAK was combined with AtUSP yielding a total of 14.8 g of UDP‐GlcA over 28 h of continuous production resulting in a productivity of 493 *g*_Product_/*g*_Enzyme._

While most instances of continuous flow biocatalysis involve immobilized enzymes,^[^ ^7^ ^,^ ^8^ ^]^ a recent example from the Elling group made use of soluble enzymes, whereby the biocatalysts were trapped within the reactor, enabling the synthesis of a selection of sugar nucleotides at impressive multigram scale (**Figure** **1**).^[^ ^9^ ^]^ The group synthesized GDP‐Man, uridine diphosphate (UDP)‐Gal, UDP‐GalNAc, UDP‐GlcA, and CMP‐Neu5Ac. This was achieved using a molecular weight cutoff (MWCO) for filtration, thus keeping enzymes within the reactor and facilitating a continuous flow approach in which unreacted starting material could be cycled back into the reaction chamber, providing a valuable uplift in overall yield and scalable access.

### 2.2 Immobilized Enzymes

**Figure 2:**
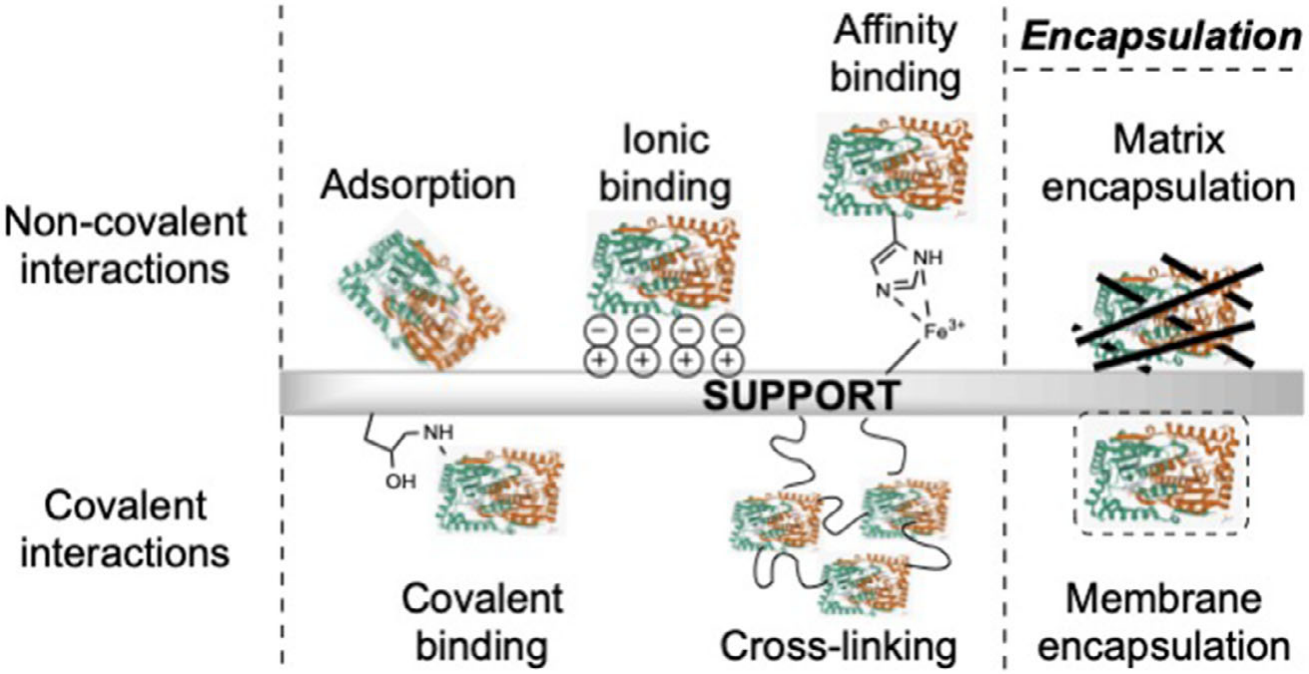
Common strategies used to immobilize enzymes including encapsulation and exploiting covalent or noncovalent interactions.

The use of immobilized enzymes is often considered advantageous due to the innate ability to recycle the biocatalyst. This is coupled with an oftentimes observed increase in stability, enabling long‐term storage and reuse, with a wealth of research being conducted in this area in recent years.^[^ ^10^, ^11^ ^–^ ^12^ ^]^ Notwithstanding this, there are challenges with immobilization design, as each strategy is often enzyme specific. Generally, this falls into one of three categories: binding to a solid support, crosslinking, and encapsulation (**Figure** **2**).

Types of solid supports that have been adopted include polymer‐based resins functionalized with different groups (e.g., amines) to enable covalent attachment of the target enzyme to the resin (**Table** **1**). This method is often a preferential choice due to the irreversibility of the support–protein conjugation. However, further consideration is becoming necessary regarding the use of such supports, given their often origin from nonsustainable sources and this has paved the way for exciting bio‐based supports to emerge, for example, making use of carbohydrate binding domains.^[^ ^13^ ^,^ ^14^ ^]^ Another common immobilization option includes coordination of protein histidine tags to a supported metal center, taking advantage of common purification tags used in immobilized metal affinity chromatography. However, such systems can suffer from enzyme leaching and loss in activity over time.^[^ ^15^ ^]^ An additional consideration when choosing an immobilization support for a process with industrial potential is cost effectiveness. While permanent binding of an enzyme to a support may well pave the way for better activity retention, the support would not be reusable. Supports making use of reversible binding types such as affinity binding could therefore be considered a more cost‐effective solution over time, with the ability to remove inactive enzyme and replace with new.

**Table 1:** Examples of selected commercial solid supports used for enzyme immobilization.

Support	Functionalization	Binding	Example reference
ReliSorb SP400	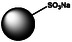	Ionic	[16]
EziG Amber	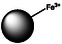	Affinity	[18]
PureCubeNi−IDA MagBeads	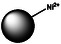	Affinity	[19]
Purolite ECR8309F	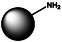	Covalent	[18]
Purolite ECR8205F	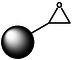	Covalent	[33]

**Figure 3:**
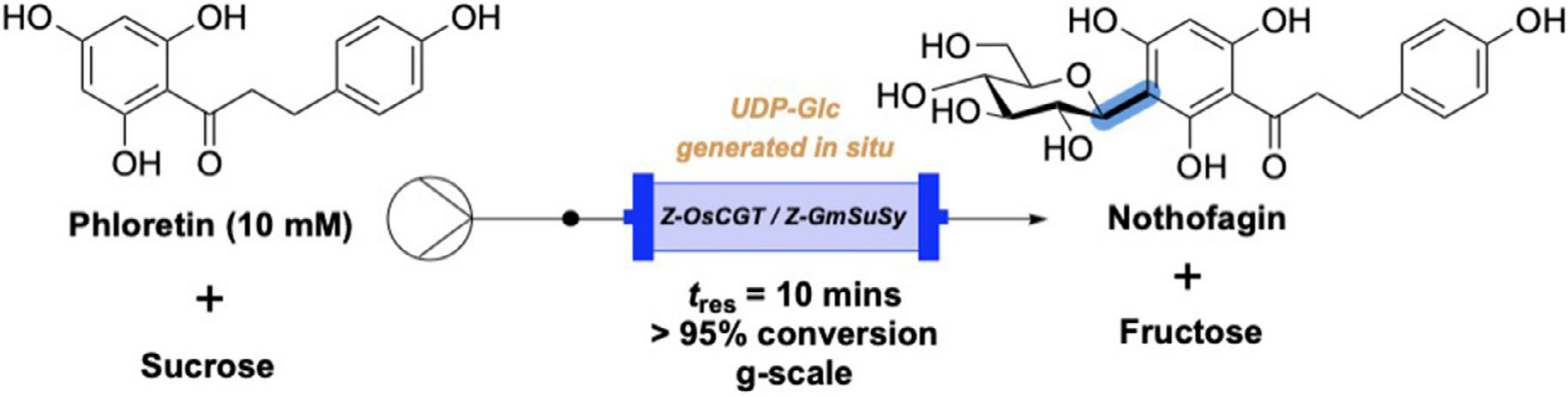
Continuous flow biocatalysis platform for nothofagin synthesis using a two‐enzyme cascade with CGT and suSy. The sucrose/SuSy combination generates UDP‐Glc in situ for the glycosyltransferase.

Nidetzky and colleagues recently developed a continuous flow system using coimmobilized enzymes in a packed bed reactor to synthesize the natural product *C*‐glycoside nothofagin on gram scale. (**Figure** **3**).^[^ ^16^ ^]^ To achieve this, two enzymes, a sucrose synthase (SuSy) and a *C*‐glycosyltransferase (CGT), were fused to a cationic binding module *Z*_basic2_. This tag facilitated immobilization onto an anionic resin, ReliSorb SP400. Due to the selective nature of the binding achieved, coimmobilization was possible directly from a mixed cell lysate, removing the need for enzyme purification. While there was some evidence that enzymes not containing the binding module were also immobilized, there was no indication that this caused a negative effect on the reaction. Once coimmobilized, the biocatalyst system was loaded into a packed bed reactor, held at 30 °C, and connected in flow, achieving conversion with 10 mM substrate to greater than 95% and a residence time of 10 min.

While coimmobilization was successful here, for systems containing one enzyme with lower thermal stability or weaker binding to the support (than the other), this may not be a suitable strategy. Compartmentalization in continuous flow, using two packed bed reactors held at different temperatures for two different enzymes, offers a solution to this issue where the enzymatic reactions are sequential; however, it would not be an option for coupled enzyme reactions. An example of successful compartmentalization was reported recently for the synthesis of trehalose in flow using monolithic microreactors.^[^ ^17^ ^]^ A UDP‐Glc pyrophosphorylase (TaGalU) and a trehalose transferase‐mCherry (mCherry‐TuTreT) were immobilized on functionalized silica supports and placed into two separate packed bed reactors. The first reactor (containing TaGalU) was held at room temperature while the second (containing mCherry‐TuTreT) was held at 55 °C. This system was capable of achieving a space time yield (STY) of up to 14.4 *g*_product _L^−1 ^h^−1 ^mg_protein_^−1^ for TaGalU and 49.6 *g*_product _L^−1 ^h^−1 ^mg_protein_^−1^ for mCherry‐TuTreT with a residence time of 10 min, and the ability to maintain steady state conversion for 100 h, which demonstrates the benefits of long‐term stability offered by protein immobilization. Further testing surrounding the stability of the immobilized biocatalyst demonstrated good activity retention after one month storage at 4 °C.

**Figure 4:**
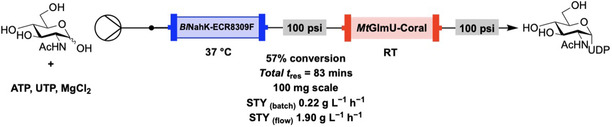
Continuous flow system utilizing a two‐enzyme cascade for the generation of GlcNAc 1‐phosphate and conversion to UDP‐GlcNAc. The system was able to retain over 50% of its initial activity over four reaction cycles.

Recently, Roberts et al. utilized a similar compartmental approach for the synthesis of UDP‐GlcNAc, where two enzymes (BlNahK and MtGlmU) were immobilized on solid supports and incorporated into a continuous flow system which was demonstrated to synthesize UDP‐GlcNAc on 100 mg scale with up to an 11‐fold increase in STY (**Figure** **4**). In this system, BlNahK was held at 37 °C while MtGlmU was held at room temperature, achieving increased biocatalyst lifetime and paving the way for continuous use of the immobilized enzymes for a further three reaction cycles (when compared to the system where both enzymes were held at 37 °C). This approach removed the need for an additional enzyme to break down the inorganic pyrophosphate byproduct (which is known to inhibit MtGlmU) via continuous removal from the packed bed.^[^ ^18^ ^]^

For most continuous flow biocatalysis systems, a simple packed bed reactor containing immobilized enzyme is used, with a reaction mixture then passing through. Relatedly however, microreactors have also been developed.^[^ ^19^ ^]^ Here, the Elling group developed a compartmentalized microreactor system for glycan synthesis using sugar nucleotide donors. Their system harnessed a total of six enzymes which were immobilized on magnetic beads to synthesize a trisaccharide target (a nonsulfated HNK‐1 epitope) in a yield of 96% which equated to a STY of 17.6 g L^−1 ^d^−1^ and was demonstrated to be 40% higher yielding than the equivalent approach using soluble enzymes (however, STY for the soluble enzyme process was not reported). The use of magnetic beads for the immobilization of each enzyme supported their transfer into the reactor and facilitated easy separation from the products. They also serve as a tool for simple separation, should each enzyme be housed in its own reactor, which could pave the way for complex enzyme systems to be individually immobilized in reactors, enabling the removal of each biocatalyst independently should one become deactivated.

### 2.3 Novel Recycling Systems

**Figure 5:**
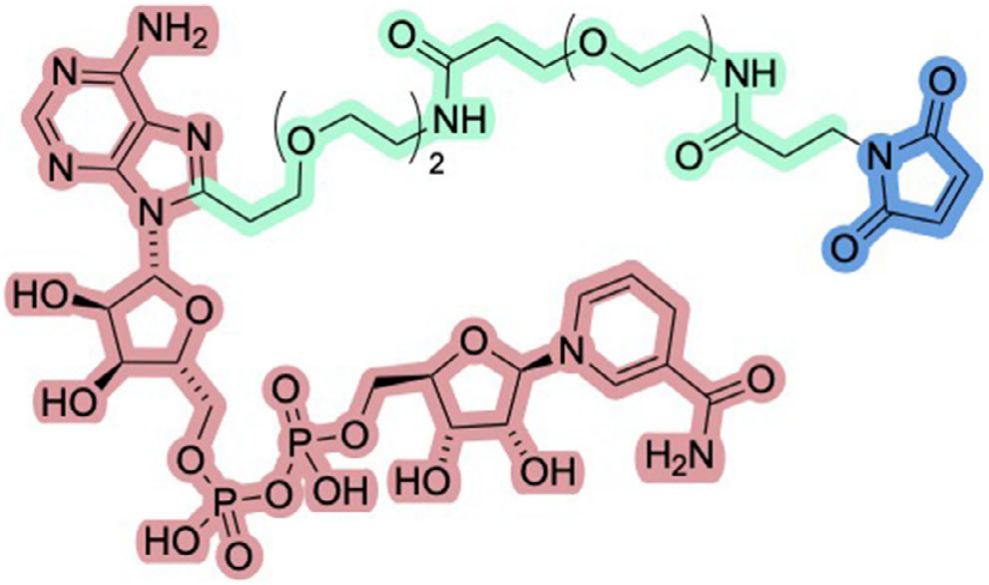
Tethering system capable of immobilizing NADH (red), linked through to malemide (blue) via a PEG‐based linker (green).

A drawback in many biocatalytic transformations, including those utilizing sugar nucleotides, is a need for expensive cofactors (e.g., NAD(H), ATP), which may limit the ultimate scalability through economic unfeasibility.^[^ ^20^ ^]^ To overcome this issue, the introduction of an additional enzyme to regenerate the cofactor has been made and while there are several such systems that are shown to work well in batch,^[^ ^21^ ^,^ ^22^ ^]^ incorporating such technology within continuous flow remains a challenge. There are examples where an enzyme for a recycling system has been immobilized and a substoichiometric amount of cofactor used with an inexpensive precursor.^[^ ^23^ ^]^ While such systems demonstrate great potential, a more ideal scenario would be for a system to be self‐sustaining, with cofactors and their recycling enzymes all tethered within the same reactor. This would also help with downstream processing and removal of waste materials from the product stream. To this end, engineering of enzymes has facilitated the fusion of three enzymes together,^[^ ^24^ ^]^ providing a system containing an enzyme to catalyze the desired reaction, an enzyme for recycling the cofactor, and a conjugation enzyme to enable covalent immobilization to a surface. Additionally, introducing recycling systems through coimmobilization of the recycling system together with the cofactor and the enzyme of interest can be achieved by the incorporation of a PEG linker and a maleimide group (illustrated in **Figure** **5** for NADH), ultimately enabling conjugation to a thiol‐based resin.^[^ ^25^ ^]^

One of the main limitations with these methods, or indeed any system with coimmobilised enzymes, is inactivation of one enzyme while the other(s) remains active. One way to overcome this, where compartmentalization cannot be used, is in the choice of resin on which the enzymes are immobilized, such as orthogonal magnetic technologies (*vide supra*).

## 3 Outlook

### 3.1 Enzyme Immobilization

The absence of a generic approach for immobilization on a solid support delivers a time‐intensive process issue of trial and error, to determine the support on which a suitable mass of enzyme can be immobilized while retaining activity for multiple reaction cycles. Paradisi and co‐workers have developed software which aims to address this issue. CapiPy is designed to analyze the surface of an enzyme with a view to suggesting the most suitable immobilization method, which, if combined with tools such as AlphaFold, could streamline the workflow of selecting the most appropriate support.^[^ ^26^ ^]^ In developing immobilization on solid support for use in packed bed reactors there is a need to consider sustainability of the materials used and pave the way for stronger, effective binding. An ideal method should preferentially bind to tags that can be introduced at either the C or N terminus of a given protein without undermining stability or activity. Carbohydrate binding modules developed by Roberts et al. and Rocha et al. offer an early insight here,^[^ ^13^ ^,^ ^14^ ^]^ for the immobilization of a transaminase and an imine reductase/glucose dehydrogenase. In the same work, Rocha et al. also trialed a silica‐binding domain which was successful in the immobilization of fluorescent proteins; however, it was unsuccessful for immobilization of an active transaminase. Increased binding to a given support would allow for direct immobilization from cell lysates to become commonplace, obviating time consuming enzyme purification.

Furthermore, encapsulation as an immobilization strategy, using metal organic frameworks or covalent organic frameworks, has been used with carbohydrate active enzymes.^[^ ^27^ ^,^ ^28^ ^]^ While yet to extend to sugar nucleotides, this could be beneficial for metal–ion cofactor dependent enzymes, expanding the scalability option for using such enzymes without the need for continuous doping with metal cofactors.

Due to the current enzyme specific nature of enzyme immobilization, one continuing obstacle for the community is the difficulty in drawing comparison between different types of immobilization. While a crude comparison between processes based on total turnover number (TTN) or STY is possible, any differences can either be attributed to the immobilization technique employed or the activity of the enzymes themselves. It is therefore challenging to compare different supports due to the difference in need for each biocatalyst. Classing supports by properties (e.g., hydrophobic or hydrophilic) could help with support selection, or further developing informatics‐based approaches, such as CapiPy.

### 3.2 Reactor Design

Access to personalized reactor design and manufacture (e.g., using a 3D printer) could pave the way for immobilized enzymes to be added to compartmentalized reactors in new ways. A potential issue with this technology is difficulty in transferring to industrial‐scale biocatalysis, which may limit applications. However, one potential solution to this has recently been developed in the form of a universal reactor which includes a removable, adaptable reaction chamber.^[^ ^29^ ^]^ This could allow for bespoke reactors in which multiple enzymes could be held in separate, removable chambers enabling a “plug and play” system where limitations such as activity loss of one enzyme in a cascade could be overcome with ease. Industrial scalability could represent a problem for such methodologies; however, with the recent uptake of flow technologies within the pharmaceutical industry, one could imagine that existing systems utilized by industry could be adapted for the large‐scale use of continuous flow biocatalysis in this form.^[^ ^30^ ^]^

### 3.3 Downstream Processing

While enzymes provide a green alternative to many synthetic challenges, downstream processing and purification can present a significant challenge. Buffer salts that may provide an optimum conversion for a given reaction can hamper purification. When this is combined with multiple cofactors, a production stream for an enzymatic process can become complex. One possible solution in flow is employing capture and release techniques, engaged recently by Tamborini and Romano,^[^ ^31^ ^]^ where a hydroxide‐based resin was used to capture the product of a biocatalytic reaction that was then released upon acidification, thus facilitating in‐line purification. Whilst there are currently no reported examples of this for sugar nucleotides, one may envisage strong anion‐exchange (SAX) to be worthy of exploration, given the charged nature of common reaction byproducts (e.g., uridine diphosphate). The complex nature of some crude product streams can further complicate purification, even with preparative high performance liquid chromatography/SAX available. This can be combated using additional enzymes to break down spent cofactors. For example, triphosphate‐containing cofactors and their di‐ and monophosphate equivalents are broken down by acid phosphatase.^[^ ^32^ ^]^

While clearly still in its relative infancy, capabilities surrounding the production and use of sugar nucleotides using continuous flow biocatalysis are developing. The number of different biotransformations that are possible and required using CAZymes will not diminish (https://www.cazy.org/), and flow biocatalysis offers an opportune technology to successfully scale up processes involving these omnipresent enzymes. It could be anticipated that with improvement in immobilization technologies, reactor systems could be designed for the in‐flow synthesis and use of sugar nucleotides in one telescoped reactor. Notwithstanding, there are several burgeoning areas that need to be overcome, representing an important multidisciplinary challenge that lies ahead.

## Conflict of Interest

The authors declare no conflict of interest.
